# Disclosing the Complexities of Childhood Neurodevelopmental Disorders

**DOI:** 10.3390/children12010016

**Published:** 2024-12-25

**Authors:** Luigi Tarani, Marco Fiore

**Affiliations:** 1 Department of Maternal Infantile and Urological Sciences, Sapienza University of Rome, 00185 Rome, Italy; 2 Department of Sensory Organs, Institute of Biochemistry and Cell Biology (IBBC-CNR), Sapienza University of Rome, 00185 Rome, Italy

Neurodevelopmental disorders represent an important and complex area of pediatric medicine, including a wide range of conditions affecting brain and nervous system functioning during development [1,2,3,4,5,6,7]. The Special Issue of *Children*, titled “**Neurodevelopmental Disorders in Pediatrics**”, assembled innovative research and clinical insights to address the challenges and advancements in the diagnosis, understanding, and management of these disorders [8,9,10,11,12,13,14,15]. In the present editorial, we summarize the contributions of the 14 published papers that together potentiate our knowledge and stimulate ongoing efforts in this crucial field.

**Scope of the Special Issue**

Neurodevelopmental disorders affect approximately 15% of children aged 3 to 17 in Western countries, displaying impairments in the behavior, learning, motor skills, communication, or other subtle neurological functions [16,17,18,19,20,21,22]. This issue aimed to cover a wide spectrum of circumstances, including but not limited to autism spectrum disorders (ASD), cerebral palsy, attention-deficit/hyperactivity disorder (ADHD), neurogenetic conditions, alcohol abuse-induced changes, communication and coordination disorders, and sensory impairments. Despite substantial progress in recent years, these diseases continue to represent therapeutic and diagnostic challenges, requiring interdisciplinary approaches that amalgamate clinical practice, research, and community support [3,4,7,23,24,25,26,27].

**Considerations from the Published Papers**

The contributions in this Special Issue show the extent and complexity of neurodevelopmental research. Key themes include:

*Advances in Diagnosis and Assessment*

Some papers focused on advanced diagnostic tools and methodologies to potentiate the early detection of neurodevelopmental disorders. For example, studies on neuroimaging, biomarkers, and machine learning algorithms offered promising opportunities for more detailed and timely diagnoses [28,29,30,31,32,33,34,35]. Such advancements highlight the importance of integrating technological novelties into routine clinical practice, aiming to reduce diagnostic delays and improve intervention outcomes.

*Intervention and Treatment Strategies*

Several articles investigated the therapeutic approaches, from pharmacological treatments to behavioral interventions and original rehabilitative treatments. Recurring emphases were on the significance of personalized and family-centered care to address the exclusive needs of each kid [10,19,36,37,38,39]. These works emphasize the requirement of combining traditional therapeutic models with emerging approaches to optimize treatment effectiveness and delivery.

*Epidemiological Insights*

A few papers presented epidemiological findings that examined the risk factors, prevalence, and outcomes associated with neurodevelopmental disorders. These works stressed the influence of environmental, genetic, and socio-economic factors, emphasizing areas for preventive actions. These papers request public health initiatives to address modifiable risk factors, thus decreasing the overall problem of these disorders [3,19,40,41,42,43].

*Impact on Families and Caregivers*

Identifying the extensive effects of neurodevelopmental disorders, several contributions examined the emotional and psychosocial challenges played by families. Interventions aimed at resilience-building and caregiver support were recognized as crucial mechanisms of comprehensive care [44,45,46,47,48,49]. These findings stress the importance of combining mental health assistance for caregivers as a key part of full treatment plans.

*Educational and Community Integration*

Data on community programs and school-based actions showed the central value of cooperative efforts to support the inclusion and development of children with neurodevelopmental conditions [50,51,52,53,54,55]. Personalized educational approaches and social skill-building proposals appeared as pivotal in fostering a helpful environment for affected kids.

**Specific Research Contributions**

The data and findings presented in this Special Issue contribute to a nuanced understanding of various neurodevelopmental disorders. Highlights include:

- The Executive Functions and Occupational Routines Scale (EFORTS) efficiently evaluates children’s executive control over daily functions, detailing both “hot” and “cool” executive functions, making it a consistent instrument for the early discovery of executive delays before prescribed schooling (Contribution 1);
- While autism spectrum disorder and social communication disorder share resemblances in pragmatic and communication troubles, some peculiarities have been recognized, e.g., in the impact on socialization and executive functioning, which may be associated with the absence of restricted and repetitive behaviors or interests in social communication disorder. These outcomes emphasize the contests posed by this nosographic separation during diagnostic assessments caused by the insufficiency of discriminative tools (Contribution 2);
- Some pathogenic mechanisms underlying the macrocephaly phenotype could be correlated with the PPP2R5D gene variants (Contribution 3);
- Sex dissimilarities in white matter diffusivity in kids with developmental dyslexia imply that girls might use a more bilateral or alternative reading system (Contribution 4);
- Categorizing the effects of proprioception on emotional–social performance in neurodevelopmental disorders and underscoring proprioception as a potential therapeutic target for harmonizing emotion regulation in kids with neurodevelopmental conditions such as autism spectrum disorder, cerebral palsy, and other different neurodevelopmental situations (Contribution 5);
- Children with 22q11.2 deletion syndrome (22q11.2DS) often deal with challenges with articulation and expressive language skills, making them at risk for developing speech-sound disorders and language impairments. Given that speech fluency plays a crucial role in cognitive functioning and healthy peer socialization, and since language changes in school-aged children with 22q11DS might signal a sharp risk for later psychotic symptoms, it is crucial to include individuals with the 22q11.2 microdeletion in early intervention programs targeting language and speech development as soon as the diagnosis is established. (Contribution 6);
- The common comorbidities of Tourette syndrome differentially affect the cognitive abilities of children and adolescents, leading to a multidimensional impact on their noticed quality of life. A longer history of tics is associated with worse cognitive outcomes, emphasizing the importance of considering disease duration in both clinical practice and research involving these affected children (Contribution 7);
- Evans’ index predicted neurodevelopmental delay in all Bayley domains in children with microcephaly associated with congenital Zika syndrome (Contribution 8);
- In a child presenting with developmental delay/intellectual disability and a craniofacial phenotype reminiscent of a Pitt–Hopkins syndrome-like condition, it was found that a novel pleckstrin homology domain-interacting protein pathogenetic variant (MIM#610954) (Contribution 9);
- Perioperative management is highlighted as vital, necessitating a multidisciplinary approach and precise surgical techniques to mitigate severe complications in newborns affected by meningomyelocele (Contribution 10);
- Treatment with Atomoxetine for attention-deficit/hyperactivity disorder symptoms in 3- to 6-year-old children emerged to be well tolerated in patients with comorbid autism spectrum disorder (Contribution 11).

**Contributions from Discussion Papers**

- The main immunological characteristics documented thus far and that likely have a role in the clinical manifestations and pathogenesis in kids affected by pediatric acute-onset neuropsychiatric syndrome (PANS) and pediatric autoimmune neuropsychiatric disorders associated with streptococcal infections (PANDAS) (Contribution 12);
- As shown in animal models, a multifactorial approach combining nutritional supplementation, antioxidant administration, behavioral techniques, and pharmacotherapy personalized to the severity and period of the disease may be an encouraging opportunity for further research in humans affected by fetal alcohol spectrum disorders (FASD) (Contribution 13);
- Despite more than a decade of evidence indicating that acetaminophen may harm neurodevelopment, research shows it is often administered to kids in dosages exceeding approved limits and under situations where it offers no clear advantage. Furthermore, findings have not clearly established any long-term advantages of acetaminophen use in pediatric populations, offering no justifiable basis for its continual use given the associated jeopardies to neurodevelopment (Contribution 14).

**Reflections and Future Directions**

This Special Issue emphasizes the multifactorial nature of neurodevelopmental conditions, emphasizing both the work still required and the progress made to improve outcomes for affected children and their families. Distinguished trends involve *i)* the growing role of technology in therapy and diagnosis, *ii)* the importance of multidisciplinary partnership, and *iii)* the requirement for complete approaches that deal with educational, medical, and social topics.

Future research should continue to investigate:

- The molecular and genetic processes underlying these conditions to better disclose targeted treatments.
- Multidisciplinary studies investigating novel developmental topics and long-term outcomes.
- Strategies to reduce gaps in the access to therapeutic and diagnostic tools, especially in underdeveloped communities.
- The proper integration of mental health services and caregiver support into routine care structures.

**Acknowledgments and Conclusions**

Of course, we extend our genuine gratitude to the authors, reviewers, and members of the editorial team whose actions contributed to this Special Issue. Their contributions have potentiated the understanding of specific topics of neurodevelopmental disorders and provided subtle data for researchers, clinicians, and policymakers.

We have confidence that the insights shared in this Special Issue could inspire persistent research, raise collaboration, and finally improve the lives of children with neurodevelopmental disorders and their families. We believe also that this Special Issue could provide a supplementary step in the study of neurodevelopmental disorders for disclosing other appropriate methods of early interventions to improve outcomes. Likewise, the findings and discussions included in this Special Issue might be of interest to professionals involved in the wide field of neurodevelopmental disorders.
